# Possible paths to increase detection of child sexual abuse in child and adolescent psychiatry: a meta-synthesis of survivors’ and health professionals’ experiences of addressing child sexual abuse

**DOI:** 10.1080/17482631.2022.2103934

**Published:** 2022-07-29

**Authors:** Signe Hjelen Stige, Ann Christin Andersen, Jorunn E. Halvorsen, Margrethe Seeger Halvorsen, Per-Einar Binder, Elida Måkestad, Ane Ugland Albæk

**Affiliations:** a Department of Clinical Psychology, University of Bergen, Bergen, Norway; b Regional Centre for Child and Youth Mental Health and Child Welfare (RKBU), Department of Mental Health, Faculty of Medicine and Health Sciences, Norwegian University of Science and Technology (NTNU), Trondheim, Norway and Møre Og Romsdal Hospital Trust, Volda, Norway; c Øyane DPS, Bergen Hospital Trust, Bergen, Norway; d Department of Psychology, University of Oslo, Norway; e Bergen Hospital Trust, Bergen, Norway; f Faculty of Health and Sport Sciences, Department of Psychosocial Health, University of Agder, Kristiansand, Norway

**Keywords:** Child sexual abuse (CSA) disclosure, uncover, detect, facilitate, health professionals, survivors, child and adolescence psychiatry

## Abstract

**Background:**

Efforts are directed both towards prevention and early detection of Child sexual abuse (CSA). Yet, only about 50% of CSA survivors disclose before adulthood, and health professionals rarely are the first disclosure recipients. Increasing the detection rate of CSA within the context of Child and Adolescent Psychiatry (CAP) therefore represents a significant secondary prevention strategy. However, facilitating CSA disclosure when the survivor is reluctant to tell is a highly complex and emotionally demanding clinical task. We therefore argue that efforts to increase detection rates of CSA within CAP need to rest on knowledge of how both survivors and health professionals experience addressing CSA.

**Method:**

Using meta-ethnography as method, we present separate sub-syntheses as well an overarching joint synthesis of how survivors and health professionals experience addressing CSA.

**Results:**

Results show how both survivors and health professionals facing CSA disclosure feel deeply isolated, they experience the consequences of addressing CSA as highly unpredictable, and they need support from others to counteract the negative impact of CSA.

**Conclusion:**

The results indicate that adapting the organization of CAP to knowledge of how the survivors and health professionals experience addressing CSA is critical to facilitate earlier disclosure of CSA within CAP.

## Introduction

There is broad agreement on the significance of uncovering child sexual abuse (CSA) as early as possible with a global research effort to uncover prevalence, detection rates, and barriers and facilitators to disclosure (Stoltenborgh et al., 2015). However, only about 50% of survivors disclose during childhood and adolescence, and 20–30% of CSA survivors never disclose (London et al., 2008; McElvaney, 2015; McGuire & London, 2020). Most children experiencing CSA know the person committing the abuse (Jackson et al., 2015; WHO, 2003), increasing the probability of delayed disclosure or non-disclosure (Kogan, 2004; Smith et al., 2000). A recent review concluded that the barriers to disclose continue to outweigh the facilitators (Alaggia et al., 2019), and a thorough meta-analysis estimated that rates of self-reported CSA are more than 30 times higher than CSA rates in official reports (Stoltenborgh et al., 2011). As many as 9% of girls and 3% of boys experience CSA involving penetration worldwide (Barth et al., 2013). Negative health consequences of CSA are extensive, including somatic illness, mental illness, risk behaviour, and early death (Briere & Elliott, 2003; Coles et al., 2015; Felitti et al., 1998; Jonas et al., 2011; Trickett et al., 2011). Therefore, any intervention potentially reducing the risk of adverse health consequences following CSA is significant.

Although disclosure is paramount to stop abuse, research also shows that children who disclosed CSA and were exposed to continued abuse after the disclosure had worse mental health outcomes in adulthood than survivors of CSA who did not disclose (Swingle et al., 2016). Some research indicates that disclosure recipients’ (i.e., the person the survivor chooses as a confidant for disclosure) responses are more strongly associated with mental health outcomes in adulthood than abuse characteristics (Jonzon & Lindblad, 2005). These findings point to the importance of how survivors are met when they disclose, and thus the potential for secondary prevention in child and adolescent psychiatry (CAP). Health professionals are trained to validate people’s experiences and flexibly respond to the situation in helpful ways. Therefore, they are qualified to serve as suitable first disclosure recipients for CSA. However, health professionals are seldom chosen as first disclosure recipients (Brattfjell & Flåm, 2019; Kogan, 2004; Lahtinen et al., 2018; Manay & Collin-Vézina, 2021). This is a paradox, as we know that the prevalence of CSA is higher among children and adolescents receiving mental health care than in the general population (Spataro et al., 2004). Thus, facilitating earlier disclosure of CSA in CAP may be a key secondary preventive strategy (Easton, 2019).

However, facilitating earlier disclosure of CSA when the survivor has not decided to tell is a highly complex clinical task. Ultimately, intentional disclosure depends on the survivor’s ability and willingness to tell and opportunities to do so (Brennan & McElvaney, 2020). A prerequisite for survivors to disclose is that they understand that their experiences fall within the category of CSA and that their experiences are consciously available to them. Population-based studies report that as many as 50% of children do not self-label their experiences as CSA (Lahtinen et al., 2018). Many survivors don’t remember and/or understand their experiences as CSA until years later due to lack of knowledge, loss for words, or underdeveloped cognitive maturity to understand CSA (Stige et al., 2020). CSA also has consequences for the ways survivors think about, identify, and understand themselves and what they believe others will think of them (Halvorsen et al., 2020; Paine & Hansen, 2002; Reitsema & Grietens, 2016). Because of the grooming process frequently involved in CSA, survivors often have ambivalent feelings towards the person committing the abuse and feel they have participated in the abuse. Survivors, therefore, frequently feel responsible for and ashamed about the abuse. They also often fear not being believed or being blamed for the abuse if they tell, and they fear negative consequences for their family and the person committing the abuse (Hershkowitz et al., 2007; Lahtinen et al., 2018; Lemaigre et al., 2017; Morrison et al., 2018; Münzer et al., 2016; Paine & Hansen, 2002). Many adolescents also use indirect disclosure strategies, like self-harming behaviours (Ungar et al., 2009). Keeping the abuse secret may also serve as a way to stay in control, avoid being overwhelmed, and maintain normality (Ungar et al., 2009). These typical reactions to abuse, illustrate the tremendous task for survivors and the complexity of the clinical judgements facing health professionals when working to facilitate disclosure of CSA.

The complexity of the child’s situation and the task at hand when attempting to disclose CSA is also reflected in the conceptualization of “disclosure”. The understanding of disclosure has changed over time, from being viewed as a discrete one-time event, to a relational process unfolding over time. While the child seeks and considers possible confidants, the adult can strive to provide the child with a safe space to disclose (Alaggia et al., 2019; Brattfjell & Flåm, 2019; Brennan & McElvaney, 2020; Flåm & Haugstvedt, 2013; Reitsema & Grietens, 2016; Ungar et al., 2009). Therefore, accessing someone to trust, expecting to be believed, and being asked are critical facilitators for purposeful disclosure (Brennan & McElvaney, 2020; Lemaigre et al., 2017; Watkins-Kagebein et al., 2019). In line with this, most survivors tell friends or family first (Brattfjell & Flåm, 2019; Kogan, 2004; Malloy et al., 2013), with a substantial group of survivors only disclosing to peers (Kogan, 2004; Manay & Collin-Vézina, 2021; Priebe & Svedin, 2008). This typical behavioural pattern has important clinical implications for facilitating early disclosure within the context of CAP, including the crucial role of the therapeutic relationship as a base for disclosure.

While we have some knowledge of how survivors experience the process of disclosing CSA and what facilitates and hinders this process, very little is known about how health professionals experience addressing CSA. This lack of knowledge is unfortunate, given the challenging clinical task at hand and the essential role health professionals play, in facilitating earlier disclosure within the context of CAP. Even thinking about CSA is challenging, highly stressful, and potentially emotionally overwhelming for most adults. Some research exploring professionals’ experiences of addressing child adversity more generally has pointed to the emotional burden of facing children’s suffering and the cruelty in what they have been subjected to. Health professionals reported feelings of inadequacy and fear of making things worse for the child when doing this work and felt they were being mean to the child when asking about adversity (Albæk et al., 2020, 2018).

Given the emotional strain experienced by health professionals in addressing child adversity and the importance of a trusting relationship and emotionally available adults to facilitate CSA disclosure from the survivor perspective, a key question is how to support health professionals to engage with potential CSA survivors in ways that facilitate disclosure. While empathic engagement with the trauma survivor is seen as a prerequisite for effective interventions, it is also recognized that the empathic engagement leaves the health professional vulnerable to vicarious trauma. The gradual exposure to clients’ trauma may lead to persistent changes in how health professionals view themselves, other people, and the world. There is also increased risk of re-experiencing and avoidance of clients’ trauma material, depressed mood, and potentially cynicism and loss of hope (McCann & Pearlman, 1990; Sexton, 1999). Not surprisingly, exposure to trauma content has been found to correlate with levels of vicarious trauma (Pearlman & Mac Ian, 1995; Schauben & Frazier, 1995). However, some research has found that work-related stressors predict levels of vicarious trauma better than exposure to clients’ trauma content (Devilly et al., 2009). This points to the role of organizational prevention of vicarious trauma (Bell et al., 2003). The significance of organizational prevention is also reflected in the emphasis health professionals put on collegial support and opportunities for sharing vulnerability and how the work affects them. These are mentioned as key buffers that enable health professionals to address child adversity more generally (Albæk et al., 2019), and CSA specifically (Helpingstine et al., 2021; Sommer & Cox, 2005).

The overview above points to the complexity of uncovering CSA and suggests the need to apply and integrate a broad range of perspectives to improve detection rates. We need to understand why disclosure processes are so challenging in order to find ways to facilitate earlier disclosure of CSA in general and within CAP in particular. Given the strong dynamics instigated by CSA and the formidable task of sharing CSA experiences, survivors cannot bear the responsibility for telling alone. Moreover, we should acknowledge that we ask a lot when we expect health professionals to contribute to uncovering CSA. We, therefore, believe that we need to learn from and integrate the available research on first-person perspectives of both survivors and health professionals, if we are to facilitate earlier disclosure of CSA within the context of CAP. It is, however, important to stress that while we argue that synthesizing the perspectives of survivors and health professionals can provide vital insight that can guide our efforts to increase detection of CSA within CAP, we acknowledge that the perspectives of CSA survivors and health professionals are grounded in fundamentally different and noncomparable experiences. Yet, we claim that by integrating both perspectives we create a better picture of how the clinical situation of addressing CSA within the context of CAP is experienced. By synthesizing these perspectives and reflecting on how they diverge and converge we may gain valuable insight.

Previous reviews and meta-syntheses have explored similar research questions concerning the survivor perspective. However, sources of data (qualitative and quantitative) and perspectives (adult and child) have often been combined (e.g., Alaggia et al., 2019; Lemaigre et al., 2017). Others have applied a more narrow focus, i.e., on factors affecting disclosure (Morrison et al., 2018), facilitators for disclosure (Brennan & McElvaney, 2020), or a more normative perspective (Watkins-Kagebein et al., 2019). There is, therefore, a lack of meta-syntheses exploring how children and adolescents experience the disclosure process, while no existing reviews explore the health professional perspective of addressing CSA. The current study aims to identify possible ways to increase the detection of CSA within CAP. This is done by integrating available knowledge about how children and adolescents experience the process of disclosing CSA with qualitative studies on how health professionals experience addressing CSA.

## Material and methods

Given the inherent challenges of generalization from qualitative research (Kvale, 1996), methods have been developed to accumulate knowledge across qualitative studies. Qualitative meta-synthesis is a scientific inquiry where research findings in completed qualitative studies are summarized, compared, and integrated to gain new insight, develop overarching meaning, and arrive at a deeper understanding of a topic (Britten et al., 2002; Sandelowski, 2012; Zimmer, 2006). We chose to use the meta-ethnographic comparative method (Noblit & Hare, 1988), as it is the most frequently used meta-synthesis method in healthcare research and allows for interpretation of qualitative findings from diverse settings and cultures into a higher-order understanding. We also drew on more systematic coding of findings and reflexive thematic analysis (Braun & Clarke, 2019).

### Search strategy and identification of relevant studies

To identify relevant qualitative studies for the meta-synthesis, the first author conducted a systematic literature search in PSYCHINFO on April 21^st^, 2021, using the search strategy specified in Table 1. Due to the language skills in the researcher team, only peer-reviewed articles in English or a Scandinavian language were included.

**Table 1.** Details on search strategy and inclusion criteria for articles to be included in the meta-synthesis.

Search strategy	Inclusion criteria
(“sex* abuse” OR “CSA” OR incest*) AND (“talk* about” OR “ask* about” OR “tell* about” OR identify* OR disclos* OR uncover* OR address* OR inquir* OR expos* OR assess* OR screen* OR interview*) AND (interview* OR experienc* OR qualitative OR narrative OR phenomenolog* OR “grounded theory” OR “thematic analysis” OR analysis OR interpret* OR “IPA” OR “focus group*”) AND A (child* OR youth* OR adolescen* OR young OR teen*) OR B (professional* OR therapist* OR “health care worker*” “mental health professional*” OR psychotherapist* OR psychologist* OR “service provider*” OR psychiatrist*)	*Inclusion criteria survivors*: experienced sexual abuse <18 years; not commercial sexual exploitation/trafficking; not homeless/runaways/street youth; not incarcerated/in prison; not sexual offenders who have been sexually abused; focus on process of disclosure or experience with disclosing CSA; qualitative research reporting quotes; interviewed before the age of 18. *Inclusion criteria health professionals*: not forensic interview /court processes; not treatment of difficulties after CSA; health professionals experience of interviewing for CSA/process of uncovering CSA; experiences of working with survivors of CSA if relevant for processes of uncovering CSA (i.e., how working with this client group impacts them as persons); qualitative research reporting quotes.

**Figure 1.**
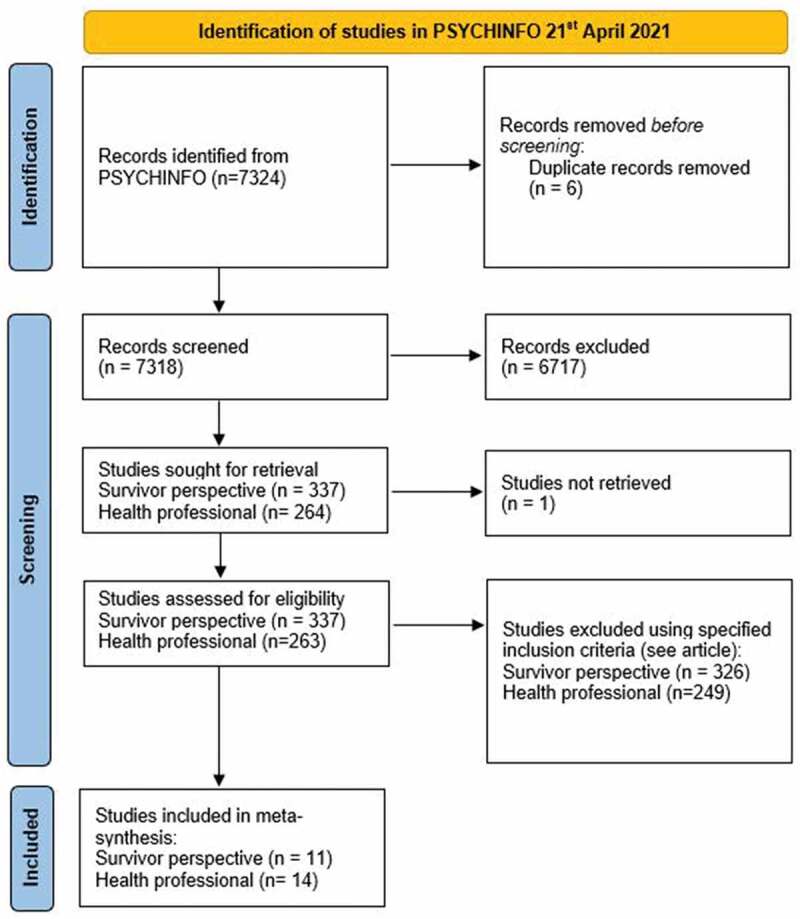
PRISMA 2020 flow diagram for inclusion of articles in the meta-synthesis.

The initial search resulted in 7318 results after duplications had been removed. Next, the first author screened all titles and abstracts to identify relevant articles using the inclusion criteria specified in Table 1. The university library was unable to access one of the articles identified in the search, and this article was therefore not retrieved. The relevant articles were then evaluated for quality using the criteria detailed by the critical appraisal skills programme (CASP, 2018). This process resulted in the inclusion of 11 articles focusing on survivors of CSA interviewed as children or adolescents and 14 articles focusing on health professionals’ experiences of addressing CSA. For details on the identification and selection of the 25 included studies, see the flow diagram in Figure 1.

The 11 included articles on the survivor perspective reported from 10 studies, including a total of 299 participants (77 male) aged 3–22 years, with studies conducted in the US, UK, Italy, Ireland, Norway, and Israel. The 14 included articles on the health professional perspective reported from 14 studies, including a total of 513 participants aged 24–68 with work experience ranging from a few months to 38 years. For 400 participants, no information on gender was provided. 11 of the remaining 113 participants were male. Studies were conducted in the US, Canada, Norway, Sweden, Ireland, Turkey, Israel, South Africa, and Australia. For an overview of the 25 articles, see appendix 1.

### Meta-ethnography and the process of synthesizing the included studies

Meta-ethnography as described by Noblit and Hare (1988) entails seven research steps; 1) isolate a research question to explore with qualitative data; 2) identify relevant studies through literature review; 3) read the studies thoroughly; 4) find how the studies are interrelated; 5) translate the studies into each other and extract overarching themes; 6) synthesizing translations; and 7) communicate findings. To safeguard the quality of the synthesis in the face of a very complex empirical material, representing two very different perspectives on the disclosure processes, synthesis progressed through three stages.

We first synthesized the studies from the survivor perspective and from the health professional perspective separately, into two sub-syntheses, before we integrated the two perspectives in our main synthesis. To support this work, particularly steps 3–5 (Noblit & Hare, 1988), the first, second, third, and sixth authors wrote summaries of each article into a live Word document in Teams, available to all team members. The online platform Miro (http://miro.com) and the template “brainwriting” were used as visual support for the analytical work. Next, the team met to synthesize the 11 studies on child and adolescent disclosure experiences. We assembled lists of concepts, themes, and key quotes from the included studies. We juxtaposed them to understand how they were interrelated while searching for findings that confirmed each other and findings that seemed to refute each other. The first author then used NVivo12 (QSR Int. Ltd, 2018) as technical support to code the relevant parts of the finding sections in the included articles. The codes were abstracted by principles of inductive reflexive thematic analysis (Braun & Clarke, 2019) and used to support an interpretive translation of studies (step 5 and 6, Noblit & Hare, 1988).

The same procedure was then used to carry out a sub-synthesis of the health professional perspective, where the creative processes of meta-ethnography were repeated. Finally, we used these two sub-syntheses as a starting point to synthesize both survivors’ and health professionals’ perspectives on the process of addressing CSA through the reciprocal translation of the themes from both sub-syntheses together with key themes and quotes from the included studies (step 6, Noblit & Hare, 1988). The fifth author acted as an external audit and was not part of the initial work on the syntheses but examined the thematic structure critically, with an intimate knowledge of the included studies.

## Results

In the following we will briefly outline the findings from the two sub-syntheses before we share the findings of the overarching synthesis more in-depth.

### Sub-synthesis of the perspectives of the CSA survivors

The sub-synthesis of the survivors’ experiences of the process of disclosing CSA resulted in the following four themes: 1)*Being part of something that cannot stand the light of day; 2)Carrying a consuming secret; 3)Finding safe ways to relieve pressure*; and 4)*The ripple effects of telling* (see, Table 2 for details on theme descriptors and articles contributing to each theme). The analysis showed how survivors went from a position of confusion regarding what they had been part of, through a process of gradually labelling their experiences as unwanted, accompanied with a growing need to share their experiences with someone. Arriving at this point, the survivor started searching for possible ways to share their experiences, including assessing potential disclosure recipients. A trusting relationship, feelings of genuine care and emotional availability proved particularly important when survivors chose disclosure recipients. Finally, after telling the survivors found themselves having to cope with the consequences of disclosure, including unexpected and negative consequences for themselves, their family, and the person committing the abuse. In this phase, some survivors questioned their decision to disclose, due to the strain of the situation.

**Table 2.** Themes resulting from sub-synthesis of the 11 included articles reporting on the survivor perspective of CSA disclosure.

Theme name	Being part of something that cannot stand the light of day	Carrying a consuming secret	Finding safe ways to relieve pressure	The ripple effects of telling
Descriptors	*Trying to figure out what happened* *Confusion and shock* *Searching for clues to understand own experiences* *Relationship to the person committing the abuse and ambivalence make it difficult to define it as abuse* *Feelings of participation induces self-blame for abuse* *Fear and inability to stop abuse induces shame* *Fearing what others would think if they knew*	*Carrying the experiences alone for a long time* *Tension between telling and not telling* *Managing the secret of CSA to protect oneself, family, and the person committing the abuse* *The corrosive effect of keeping the secret*	*Assessing potential disclosure recipients and situations to decide who and when to tell* *Continuing to manage the secret by monitoring who gets to know what* *Shared focus on CSA through TV shows or conversation makes it easier to tell* *Genuine care and interest in well-fare of survivor making room for telling* *A safe relationship and reciprocal sharing make it easier to tell*	*Dealing with the unpredictable consequences of disclosure* *Disclosure may end abuse* *Disclosure enables support from other abuse survivors* *The risk of disclosure ricocheting* *Dealing with losing control of who knows about the abuse* *Dealing with life changes brought about by the disclosure* *Dealing with consequences of disclosure for other people, including the person committing the abuse*
Articles contributing to the themes	Allnock & Atkinson, 2019 Crisma et al., 2004 Foster, 2017 Foster & Hagedorn, 2014 Jensen et al., 2005 Katz & Hamama, 2017 McElvaney et al., 2014 Shalhoub-Kevorkian, 2005 Staller & Nelson-Gardell, 2005	Crisma et al., 2004 Foster, 2017 Foster & Hagedorn, 2014 Jensen et al., 2005 Katz & Hamama, 2017 McElvaney et al., 2012 McElvaney et al., 2014 Shalhoub-Kevorkian, 2005 Staller & Nelson-Gardell, 2005	Allnock & Atkinson, 2019 Crisma et al., 2004 Foster, 2017 Foster & Hagedorn, 2014 Jensen et al., 2005 Katz & Hamama, 2017 McElvaney et al., 2012 McElvaney et al., 2014 Petronio et al., 1996 Shalhoub-Kevorkian, 2005 Staller & Nelson-Gardell, 2005	Allnock & Atkinson, 2019 Crisma et al., 2004 Foster, 2017 Foster & Hagedorn, 2014 Jensen et al., 2005 Katz & Hamama, 2017 McElvaney et al., 2014 Petronio et al., 1996 Shalhoub-Kevorkian, 2005 Staller & Nelson-Gardell, 2005

### Sub-synthesis of the perspectives of the health professionals

The sub-synthesis of the health professionals’ experiences of addressing CSA resulted in the four themes 1)*Engaging in work that stains; 2)Unexpected rewards; 3)Handling uncertainty—what will happen when you unleash the beast*?; and 4)*The need for support and self-care* (see, Table 3 for details on theme descriptors and articles contributing to each theme). The analysis showed how the work was associated with strains that had an impact on the health professionals, and that changed the way they saw themselves, others, and the world, and how they related to others. However, the work also provided unexpected rewards that gave their work meaning. They were, however, battling with the unpredictability of addressing CSA, and needed support and self-care to be able to do their work over time.

**Table 3.** Themes resulting from sub-synthesis of the 14 included articles reporting on the perspective of health professionals on addressing.

Theme name	Engaging in work that stains	Unexpected rewards	Handling uncertainty—What will happen when you unleash the beast?	The need for support and self-care
Descriptors	*Feelings of isolation—others cannot and will not understand* *Relationship to others changes* *Unwanted changes in oneself, including emotional, somatic, and trauma-related problems* *Darker view of the world and other people* *The feeling of safety is challenged* *Engagement for work is drained*	*The empowerment in being able to help someone in need* *The honour of being the one who gets to hear the story* *Expanding perspectives and gaining wisdom* *Growing as persons* *Becoming a better therapist* *Increasing engagement and activism*	*The fear of making it worse* *The tension between the wish to be spared and the obligation to see CSA* *Uncertainty regarding how to get to the real story of what has happened* *Uncertainty regarding one’s own and other’s reactions/lose control of the story* *Uncertainty regarding the system’s ability to follow up in a helpful manner*	*Balancing connection with the story and distance to avoid being overwhelmed* *Needing to feel that one is not on one’s own* *Self-awareness and to monitor the impact of the work* *Self-care necessary to counteract the draining character of the work* *The need for supervision and professional input to keep going* *The need for realistic workload and definition of tasks for work to be sustainable*
Articles contributing to the themes	Benatar, 2000 Capri et al., 2013 Jensen et al., 2010 Possick et al., 2015 Sivis-Cetinkaya, 2015 Slane et al., 2018 Steed & Downing, 1998 Sui & Padmanabhanunni, 2016 Wheeler & McElvaney, 2018	Benatar, 2000 Capri et al., 2013 Possick et al., 2015 Sivis-Cetinkaya, 2015 Steed & Downing, 1998 Sui & Padmanabhanunni, 2016 Wheeler & McElvaney, 2018	Benatar, 2000 Capri et al., 2013 Engh Kraft et al., 2017 Gallop et al., 1995 Jensen et al., 2010 Sekhar et al., 2018 Sivis-Cetinkaya, 2015 Søftestad & Toverud, 2013 Överlien & Hyden, 2003	Benatar, 2000 Capri et al., 2013 Engh Kraft et al., 2017 Gallop et al., 1995 Possick et al., 2015 Sivis-Cetinkaya, 2015 Slane et al., 2018 Steed & Downing, 1998 Søftestad & Toverud, 2013 Överlien & Hyden, 2003 Wheeler & McElvaney, 2018

### Overarching synthesis—integrating the survivors’ and the health professionals’ perspectives

The analytic process of synthesizing the perspectives of the survivors and the health professionals described above resulted in three overarching themes: 1) *The isolating effect of carrying something others cannot bear*; 2) *The unpredictability of making CSA a shared focus of attention*; and 3) *Needing to counteract the destructive impact of CSA*. For details on how descriptors from the two sub-syntheses feed into these three themes, see, Table 4. Each theme, and how it reflects both the survivors’ and the health professionals’ experiences, is detailed below.

**Table 4.** The three themes resulting from synthesizing survivor and health professional perspective on uncovering CSA, with descriptors from the themes in the two sub-syntheses.

Theme name	The isolating effect of carrying something others cannot bear	The unpredictability of making CSA a shared focus of attention	Needing to counteract the destructive impact of CSA
Descriptors from the two sub-syntheses contributing to the themes	Survivor descriptors: *Trying to figure out what happened* *Feelings of participation induces self-blame for abuse* *Fear and inability to stop abuse induces shame* *Fearing what others would think if they knew* *Carrying the experiences alone for a long time* *Managing the secret of CSA to protect oneself, family, and the person committing the abuse* *Tension between telling and not telling* Health professional descriptors: *Feelings of isolation—others cannot and will not understand* *Relationship to others changes* *The tension between the wish to be spared and the obligation to see CSA*	Survivor descriptors: *Assessing potential disclosure recipients and situations to decide who and when to tell* *Continuing to manage the secret by monitoring who gets to know what* *Dealing with the unpredictable consequences of disclosure* *Disclosure may end abuse* *Disclosure enables support from other abuse survivors* *The risk of disclosure ricocheting* *Dealing with losing control of who knows about the abuse* *Dealing with life changes brought about by the disclosure* *Dealing with consequences of disclosure for other people, including the person committing the abuse* Health professional descriptors: *The fear of making it worse* *Uncertainty regarding how to get to the real story of what has happened* *Uncertainty regarding one’s own and other’s reactions /losing control of the story* *Uncertainty regarding the system’s ability to follow up in a helpful manner*	Survivor descriptors: *The corrosive effect of keeping the secret* *Relationship to the person committing the abuse and ambivalence make it difficult to define it as abuse* *Feelings of participation induces self-blame for abuse* *Fear and inability to stop abuse induces shame* *Dealing with the unpredictable consequences of disclosure* *Genuine care and interest in well-fare of survivor making room for telling* *A safe relationship and reciprocal sharing make it easier to tell* Health professional descriptors: *Unwanted changes in oneself, including emotional, somatic, and trauma-related problems* *Darker view of the world and other people* *The feeling of safety is challenged* *Engagement for work is drained* *The empowerment in being able to help someone in need* *The honour of being the one who gets to hear the story* *Expanding perspectives and gaining wisdom* *Growing as persons* *Becoming a better therapist* *Increasing engagement and activism* *Balancing connection with the story and distance to avoid being overwhelmed* *Needing to feel that one is not on one’s own* *Self-awareness and to monitor the impact of the work* *Self-care necessary to counteract the draining character of the work* *The need for supervision and professional input to keep going* *The need for realistic workload and definition of tasks for work to be sustainable*

### Theme 1: the isolating effect of carrying something others cannot bear

#### The contribution of the survivor-perspective to theme 1: the loneliness of experiencing a secret pain

Living through experiences of CSA many survivors had instantly felt something was wrong. Still, they often battled for a long time, trying to understand what they had been part of: “All these thoughts were going through my head. Like why was he touching my chest? Why me? What just happened?” (Foster & Hagedorn, 2014, p. 545). Many survivors experienced that somebody they knew took advantage of their relationship during the abuse. This made it particularly difficult to grasp what they had been exposed to: “Why do you call it abuse? This is my father, not a criminal, and he loves me. I knew he was doing wrong things to me, but he is my father” (Shalhoub-Kevorkian, 2005, p. 1274).

Even after realizing that they had been exposed to CSA, survivors struggled with questions, like “Why was I letting him do this to me?” (Foster & Hagedorn, 2014, p. 545). Thus, many survivors felt complicit in their own abuse: “He always succeeded in convincing me, even when I said to myself before that I don’t want to; when I saw him, I couldn’t say no” (Katz & Hamama, 2017, p. 3657). The relationship to the person committing the abuse along with the physiological responses during the abuse also made it difficult to sort out whether it had in fact been abuse and whether they were partly responsible for what had happened:

I feel so ashamed even if I didn’t do anything, but … that is … well … even now I feel as if it was my fault. I don’t know why, but I wonder if I did anything, maybe he misunderstood something. (Crisma et al., 2004, p. 1040)

Feeling ashamed and guilty for not having done enough to stop the abuse made it almost impossible for survivors to tell others about their experiences: “I just didn’t want anyone to know. I was so angry with myself, ashamed with myself that it would happen again” (McElvaney et al., 2014, p. 936). Realizing the forbidden nature of the abuse meant survivors also could foresee the risks of exposing the abuse:

When I was 9 years old, my father started to touch various parts of my body … Later on, however, he started playing more and more with my body. He did it only when Mom was away, and I kept it a secret from her. No one would tell Mom about such a thing. She will get angry, punish, and hit me … and maybe fight with my father. As a child, I never wanted to lose my father; he is a very nice man. (Shalhoub-Kevorkian, 2005, p. 1274)

Some survivors also shared how the person committing the abuse would stress the negative consequences of disclosure to keep them from telling:

And then there was one time he had a talk with me. He said: ‘You must never tell anyone, it’s our secret, and if anyone finds out, I have to go to jail, and that’s the worst thing that could happen to anyone and then I’ll get beaten up in jail.’ He said all this stuff. And then it wasn’t very tempting to tell. After that it took even longer before I could tell. (Jensen et al., 2005, p. 1405)

The survivors were hence stuck in a lonely, unbearable position of not daring to tell, yet longing to tell to relieve their pain and stop the abuse:

I always wanted to tell someone. I remember going into her bedroom and leaving a note I used to wrap it up in her nightie. And then I’d think “No I can’t tell” and I’d run in and get it back. I did that loads of times. (McElvaney et al., 2012, p. 1164)

#### The contribution of the health professional-perspective to theme 1: The loneliness of hearing something others do not want to know about

The health professionals too, having opened their eyes to CSA, felt isolated. Regardless of the emotional burden of relating to CSA, they found themselves unable to share their experiences with others outside of work: “Their first reaction is usually: ‘Children are prone to lying’. People don’t want to hear any of this. It makes one feel lonely. If I want acknowledgment, I have to find friends with the same heart” (Capri et al., 2013, p. 371). This made it difficult even to discuss the positive aspects of their work with others: “And that has an impact as well. Maybe in terms of not talking about the positives as much as one should or one would like to, or maybe not expecting that people will understand that part” (Wheeler & McElvaney, 2018, p. 518). Some health professionals also shared that they felt judged by colleagues for wanting to work with CSA, strengthening their sense of alienation:

I think colleagues are judgemental about this. I think that some people think “Yuck, who wants to work with trauma?” People don’t want to hear about it and the oppressors want to silence it. So the person working in this is causing the person to look, causing the person to see, that’s a specialty worth specializing in. And I think colleagues get a little bit anxious around that and you know, they’ll try to change the subject or they’ll try to ask a more general question or divert it somehow. (Benatar, 2000, p. 18)

Consequently, some health professionals avoided any discussion of their work: “I just say I’m a teacher. It triggers a discussion … it’s not a pleasant thing we work with. You have emotions that you can’t share because they won’t understand. That’s what supervision is for” (Capri et al., 2013, p. 371). Health professionals too found themselves avoiding CSA: “You find what you want to see, you don’t look for what this might stand for, do you? Many times, seeing it is tough, although you don’t realize you are resisting” (Engh Kraft et al., 2017, p. 138). Even though they wanted to keep their eyes open to CSA, health professionals struggled with a tension between wishing to be spared for the burdens of the work, while at the same time feeling obligated to do the work:

I think that part of me just doesn’t want to go. You know, what am I going to hear today? You know, you hear these sick stories and you think “Ach G-d, I don’t want to.” (Benatar, 2000, p. 17)

In some ways the loneliness experienced by survivors and health professionals cannot be compared, as they stem from such fundamentally different perspectives and are grounded in deeply differing experiences. Yet, our analysis showed how the survivors and the health professionals shared the experience of how actively relating to CSA, a phenomenon that others could not bear to take in and did not want to relate to, made them feel isolated and alone. Naturally, this feeling was particularly prominent for the survivors. But the health professionals too experienced that entering the field of CSA isolated them from others—even making others shun them. This left both survivors and health professionals in an utterly lonesome position, where the weight of carrying this burden alone threatened to consume them.

### Theme 2: the unpredictability of making CSA a shared focus of attention

#### The contribution of the survivor-perspective to theme 2: Both not being believed and being believed poses risks for the survivors

For the survivors, the initial concern was to find a way to tell that maximized the chance of being believed and minimized the risk for themselves and others: “I think the main fear of most people was not being believed that’s … the biggest one” (McElvaney et al., 2014, p. 934). Consequently, they spent a lot of time and energy assessing potential disclosure recipients, searching for someone whom they trusted would have room for their story and could manage it without panicking or sharing it indiscriminately with others:

G: But they believed me right away, and he admitted it, so that really helped me a lot. I can’t even imagine what it would have been like if he had lied and said he didn’t do anything.

I: Were you surprised that your friend and mom believed you?

G: No I wasn’t surprised. I was counting on that. If I had thought that they wouldn’t believe me, then I wouldn’t have said anything.

I: You wouldn’t have said anything then?

G: No it would have made everything worse really. You experience a lot of shit, then you tell about it, and they don’t believe you, and think of you as a liar. (Jensen et al., 2005, p. 1406)

Finding someone to confide in was, however, immensely difficult, and many survivors regarded themselves lucky to have done so: “The problem is there’s only a slight few friends you can confide in and I was lucky I found two” (McElvaney et al., 2012, p. 1165). The unpredictability of responses and consequences also meant disclosing was a process. The survivors continued to monitor the responses of the persons they shared their story with and what was shared with whom, also after the initial disclosure of the abuse: “I think I hinted around the point a little bit and then it just—the hinting feeling got to the point where I felt that I could trust her” (Petronio et al., 1996, p. 192).

For some survivors, their fears were not justified as the people they told managed their story wisely, cared for them, and ensured that the abuse stopped: “I felt safe after I told because it wasn’t going to happen anymore” (Foster, 2017, p. 864). However, for many survivors, the consequences of telling remained unpredictable, despite their best efforts to ensure a safe haven for their story. Even though they anticipated disbelief as a possible response to telling, the survivors were profoundly affected when it became true:

I mean, the whole idea just pisses me off that here I was I couldn’t have been like, seven or eight, why would I lie about something like that? I don’t see stuff like that on TV, when I was watching Scooby Doo then. Scooby Doo didn’t teach me that. You know, I wasn’t going to lie about it and I didn’t. They didn’t believe me, and it was just pathetic. (Staller & Nelson-Gardell, 2005, p. 1423)

Moreover, the survivors also found themselves having to deal with consequences far beyond what they had anticipated, including involvement of official services and demanding investigations:

Then I had to go tell the guidance counselor and officer [blacked out name]

and a bunch of other people I did not know at all … He [the school

resource officer] told me that I had to fill out a statement about all the times

that it happened and add ALL the details. (Foster & Hagedorn, 2014, p. 547)

When disclosure caused the abuse to end, naturally the survivors felt relieved. However, revealing the abuse also started a chain-reaction of events imposing life-changes for both the survivor, the survivors’ family, and the person committing the abuse. Many survivors thus felt as if telling had caused them to lose their family, and struggled with the consequences of the disclosure:

Like when I was six I was being sexually abused by my uncle, and then one day—because my mom, she was never home, right—so then one day when she came home and my uncle was out, I told her. And then, after that they put, um me in foster care and my other sisters and brothers. I’ve been in like, fourteen or more different foster homes, one after another. It’s hard when you switch different schools. (Staller & Nelson-Gardell, 2005, p. 1424)

In some survivors the cease of contact with the person committing the abuse elicited a process of grief, because they knew and cared for this person:

Girl 2: How to deal with missing somebody that was always there for you. Well at least that’s how I feel, I mean I know that’s not a lot to it but that’s how I feel.

Moderator: I think probably that is true for lots of people if it was someone that they trusted and cared about all of a sudden that person is not there.

Girl 2: It’s almost like a death. (Staller & Nelson-Gardell, 2005, p. 1424)

The outcome of disclosing CSA was thus ambiguous for many of the survivors, irrespective of whether their first experience with telling was good: “I don’t know em there’s a part of me says that I’m glad I told but there’s another part of me says that I shouldn’t have because I split up the family” (McElvaney et al., 2014, p. 936).

#### The contribution of the health professional-perspective to theme 2: The uncertain consequences of uncovering CSA

For the health professionals, previous experiences of unwanted consequences for the survivors after disclosure sometimes lead them to think twice about bringing up CSA. This might both consciously and unconsciously lead to avoidance and could delay the process. However, their main concern was how to access the story without causing further harm—and how to ensure that sharing the story became part of a healing process. Thus, health professionals believed that sharing their story had both harming and healing potential for the survivor. The challenge was the unpredictability in how to minimize harm and maximize healing. Integrated in this perspective was the health professionals’ understanding of CSA disclosure as a process, where the survivors had to be equipped to bear their own story if harm was to be avoided: “Often people have to be ready to discuss these issues and sometimes ‘pushing’ people to talk can make it worse” (Gallop et al., 1995, p. 149). Asking at the wrong time could, then, bring up extremely strong reactions in the survivor:

17 T but we did ask her (.) or rather we said we realized (.) like that the girls’ contact person she was like in her black books for two months after that and so was I you know

18 now

18 CO¨ Aha

19 T because I was with her that time

20 C¨O You told her you suspected it

21 T Yes and then she wanted to do us (.) from a psychological point of view she wanted to murder us then (.) when we said we knew. (Överlien & Hyden, 2003, p. 229)

However, their understanding of and respect for the survivor’s own disclosure process included an understanding of how hard it was for the survivor to be the one to bring up CSA: “I have never had a situation where a pupil has come and given a direct account of what they have been subjected to” (Engh Kraft et al., 2017, p. 137). Health professionals thus felt a responsibility to introduce CSA in the conversation. Still, being the one introducing CSA also felt risky for the health professionals. On the one hand they were concerned about swaying the child, producing false memories or obstructing legal processes:

One is afraid of putting words into the mouth of the child … of using words they don’t know, right? For instance, to say ‘willy’ and ‘butt’ and ‘to stick’ … I mean, these are words you are afraid of using when talking with children. (Søftestad & Toverud, 2013, p. 1515)

On the other hand, health professionals were afraid of losing their ability to help the child if they moved too quickly or pushed too hard. They were therefore struggling to find the balance between uncovering potential CSA, which was uncomfortable for the child, while also giving sufficient room for positive things: “I felt there was a fine balance between talking about emotional themes and focusing on other things, between letting him take charge and taking charge myself” (Jensen et al., 2010, pp. 470–471). However, bringing CSA into focus and exposing abuse was also associated with a risk of losing contact with the child due to strong reactions from people around the child (see e.g., Engh Kraft et al., 2017, p. 136). Finally, uncertainty regarding CSA disclosure was immersed in feelings of helplessness regarding how the system would treat the survivor and handle the situation once they had uncovered CSA and reported it: “The biggest frustration is the system, and there’s nothing you can do … welfare, police, courts, jails, schools, parents who don’t have insight” (Capri et al., 2013, p. 372).

As we see in this theme, both the survivors and the health professionals looked for ways to make CSA a shared focus of attention. This included being attuned to situational and nonverbal signs that CSA could be addressed, as well as telling/probing directly for CSA. However, both parties did so, with respect and apprehension for the strong forces unleashed by focusing on CSA, and with a strong awareness of the unpredictable consequences of doing so. Both the survivors and the health professionals hence found themselves walking on eggshells, weighing and assessing the situation and the other, to find safe ways to introduce the topic of CSA. Moreover, they were painfully aware that even with every precaution taken, it was easy to lose control of the story once it was out in the open. The anticipated and experienced outcomes of disclosure were unpredictable and partly uncontrollable for both parties. Thus, both the survivors and the health professionals felt that the stakes of making CSA a shared focus for attention were high, due to substantial uncertainty and potential for destructive outcomes. Yet, they felt an urge to do so. Both parties therefore did what they could to minimize harm, while dealing with the uncertainties of CSA disclosure the best they could.

### Theme 3: needing to counteract the destructive impact of CSA

#### The contribution of the survivor-perspective to theme 3: mending isolation and mastering pain

For the survivor, the most urgent need was to reduce the immense feeling of isolation brought about by the abuse. They needed to share the burden with somebody else: “I should find someone to talk to frankly, openly, even if this person says nothing … but just to make him or her see how I feel, all this shame and pain” (Crisma et al., 2004, p. 1043). A close relationship and others’ expressions of genuine care eased their isolation into a position where healing could start: “Like she knew by me face like there was something up. She was saying ‘tell me.’ I was saying ‘God no way no’” (McElvaney et al., 2012, p. 1162). Support from someone who cared and understood was hence crucial to turn the tide:

I had a teacher at my school … and she was like date raped and stuff. So she like definitely knows what I’m going through and we just talked about it … it’s easy to talk to her now. She knows what I’m talking about and she’s just not sitting there listening and acting like she knows what I’m saying. (Staller & Nelson-Gardell, 2005, p. 1425)

This also enabled the ones helping the survivors to sort out what they had been a part of: “The more they told me and explained and you know told me how big a deal this was the more I kind of understood and just changed me whole view” (McElvaney et al., 2014, p. 939).

In addition to mending the feeling of isolation, survivors also needed help to battle the corrosive effects secrecy had had, and the symptoms brought about by the abuse: “It happens sometimes in school and prevents me from actually paying attention because instead of learning and listening to what the teacher is saying. I just sit there and think of nothing but the abuse” (Foster, 2017, p. 862). The survivors also needed help dealing with life-changes brought about by the disclosure process:

“When is Daddy coming up?” And I just couldn’t take it … ’cos they were all like … “I want Daddy in the house like I love him where’s Daddy?” and I felt real I felt depressed like I felt like crying all the time. (McElvaney et al., 2014, p. 937)

With the complexity of their experiences, including feeling like an accomplice to the abuse and their ambivalent relationship to the person committing the abuse, many of the survivors were prepared for healing to take time and they felt that they would always carry the scars of abuse with them:

I mean every one of us is scarred for life. Whether we’re successful in the future or not, that memory is always going to be with us … I mean it leaves so much pain and so many mixed emotions inside of you that you … can’t even explain how much they feel in a life time. I mean it’s just so much that goes on that people just don’t understand and you can’t really express everything that you have to say or feel because a lot of it is still hidden inside of you. I still have a lot of feelings and emotions inside of me that I don’t even understand or don’t even feel at this point. But as I grow, I mean, they are going to come along. So, I mean it’s, it’s like a chain reaction. It’s never finished, never. Nothing is ever fully brought about or you know, fully explained, I mean, you question a lot as you go through. (Staller & Nelson-Gardell, 2005, p. 1426)

#### The contribution of the health professional-perspective to theme 3: the need for hope and belongingness

The health professionals too were struggling with the negative effects of CSA. They felt that the work robbed them of their safety: “Sometimes I don’t feel safe, even in my own environment” (Steed & Downing, 1998, no pagination). Opening their eyes to CSA left them with a darker view of the world and other people: “It [the work] is very dirtying. It suddenly colored the world for me, and it was divided into two—victims and attackers” (Possick et al., 2015, p. 822). It also changed the health professionals’ relation towards themselves and others: “A lot of the time I’m struggling with how I feel about myself and who I am” (Steed & Downing, 1998, no pagination). The health professionals too were carrying abuse with them: “You can’t get it out of your head. This stuff gets stuck and influences your life” (Capri et al., 2013, p. 374). A lurking hopelessness also threatened their work engagement and their work identity.

Health professionals were aware of these dangers, and actively attempted to counteract them with self-care:

I feel that the place where I work is filled with poisons, filled with many more things, but filled with the poisons of difficult things that I must clean myself and purify myself of after all of the radioactive materials that I encounter. (Possick et al., 2015, p. 823)

Many had made commitments to themselves to be aware of how the work impacted them: “When I first started working in this area I made a commitment to being aware of my own responses and what I react to” (Steed & Downing, 1998, no pagination). However, the necessity of self-awareness for keeping the destructive impact of CSA at bay was striking:

As a therapist, you have to be on guard all the time, to know what this is doing to you and where it is taking you. If you are not able to make separations, you yourself can be a victim of the therapy. (Possick et al., 2015, p. 821)

Health professionals were clear that self-awareness and self-care was not sufficient to make the work sustainable. They were dependent on help from others, both in the form of professional perspectives and input from training and courses, and in the form of feeling belongingness to a professional community where they could receive supervision and share their experiences with colleagues. The health professionals stressed the importance of this professional belongingness to counteract the sense of isolation working with CSA brought about: “I don’t think one can do this work without supervision. You’ll burn out. I also made an appointment with a psychologist” (Capri et al., 2013, p. 378).

While the benefits of self-awareness, self-care, training and supervision was anticipated by health professionals, they also experienced that the efforts to keep the destructiveness of CSA at bay was facilitated by unexpected rewards of the work:

As you go along, you grow, and you change constantly … working with clients changes your worldviews … I learnt about how the most broken had the greatest capacity to give and to feel and to share and … I’ve come to realise that there is always hope, no matter how difficult things may seem … I sometimes do lose hope but only for brief moments and then I look at the things that are working for me rather than what’s not working. (Sui & Padmanabhanunni, 2016, p. 131)

The experienced rewards of meeting and working with CSA survivors and the opportunity to make a difference was, then, in many ways what swayed the health professionals to continue working with CSA, despite the tension they felt between wishing to be spared and their obligation to see:

Even though many times I was tempted, to leave or not to leave, it was clear to me that I am staying in this field. That’s the power. As a person I improved myself in a meaningful way. I developed myself. It demanded of me to make an effort to the edge of my abilities and to develop myself with them. It demanded of me to also encounter the bad in me. I am today a much richer and fuller person. I wouldn’t switch the work. (Possick et al., 2015, p. 829)

The findings show how both the survivors and the health professionals had felt the destructive impact of CSA and how it changed them. They expressed a need for, and acted, to find ways to counteract these destructive forces and to avoid being crushed by their impact. Survivors needed to rebuild what the experiences of abuse and secrecy had destroyed. Health professionals had to be focused on CSA to be able to see it, but they needed to protect themselves by making room for something more than the dark world of CSA to be able to endure the work over time. Despite intense efforts from both parties to counteract the impact of CSA on their own, both survivors and health professionals were clear that this was not something anyone could do alone. Support from other people was needed to keep the destructive forces of CSA at bay.

## Discussion

The synthesis presented above shows how both the survivors and the health professionals felt profoundly isolated from others in attempting to address CSA. They also felt apprehensive addressing CSA due to the unpredictable consequences of doing so and needed help from others to counteract the destructive impact of CSA. The results of the synthesis are in line with and nuance knowledge of how survivors of CSA experience the disclosure process (Alaggia et al., 2019; Brattfjell & Flåm, 2019; Brennan & McElvaney, 2020; Ungar et al., 2009), while shedding light on how important it is to acknowledge and be attentive towards the impact work to detect CSA has on health professionals, and how we can help them face this demanding clinical task. Thus, it is important that health services take responsibility to prevent vicarious trauma in health professionals and ensure them a sustainable work situation to facilitate CSA disclosure (Bell et al., 2003). In the following we will briefly discuss the results of the synthesis in relation to existing research and clinical experience, and discuss implications of the findings for possible ways to increase detection rates of CSA within CAP.

In line with current conceptualization of disclosure of CSA as a relational process (Alaggia et al., 2019; Brattfjell & Flåm, 2019; Brennan & McElvaney, 2020; Ungar et al., 2009), our sub-synthesis of survivors’ experiences showed that disclosure was an individual process for these participants, progressing in different phases associated with unique challenges. Complementing previous meta-syntheses on child and adolescent disclosure of CSA (e.g., Brennan & McElvaney, 2020; Lemaigre et al., 2017; Watkins-Kagebein et al., 2019), our sub-synthesis shed light on the survivors’ experiences in the initial stages of disclosure. At this stage the process seemed more internally directed, as the survivors tried to make sense of what they had experienced, and their own role in the abuse. Over time, as the survivors started to understand their experiences as abuse, they increasingly found themselves torn between keeping the secret to protect themselves, their families, and the person committing the abuse, and telling others to receive help to handle the isolation and the corrosive effects brought on by CSA. In this phase of the process the focus became more outward-directed, as survivors were actively seeking a confidant that cared and would believe them, as well as a situation that allowed them to address CSA. The tension between telling and not telling and the importance of others’ responses in this phase reflect the findings in previous meta-syntheses of child and adolescent disclosure of CSA (Brennan & McElvaney, 2020; Lemaigre et al., 2017). Our sub-synthesis expands this focus by shedding light on how disclosure is not the endpoint of the process, and how the survivors are highly vulnerable in the phase following disclosure. In this respect, our findings are in line with Watkins-Kagebein et al.’s (2019) meta-synthesis of children and adolescent’s experiences of CSA and elaborate their findings by illustrating a range of possible consequences of disclosure beyond the legal system. Many survivors found themselves having to deal with unexpected and negative consequences from disclosure, both for themselves and for people they cared about, and that made some of them question their decision to disclose.

This illustrates how a trusting relationship and experiences of benevolence and genuine care are at the core of facilitative conditions for child and adolescent disclosure of CSA. Transferred to a clinical context, this should imply that the opportunity to develop a therapeutic relationship over time is essential in facilitating earlier CSA disclosure within CAP. Given the individual quality of the disclosure process, health professionals’ ability and opportunity to tailor assessment to the individual client also seems paramount. This has implications both for how we think about the type of clinical competence needed to facilitate disclosure (i.e., “appropriate responsiveness”; Hatcher, 2015), and for organization of services (i.e., flexibility in procedures and clinical autonomy). Clinical experience shows, for example, that the opportunity to meet the child or adolescent over time often is challenged by the services’ focus on productivity, high turnover, and high caseloads. In addition, procedures often require health professionals to screen for CSA with standardized measures during one of the first sessions at the clinic, before the therapeutic relationship has been established. While the findings in both this synthesis and previous syntheses on child and adolescent disclosure (Brennan & McElvaney, 2020) point to how opportunities to tell as well as probing for CSA can facilitate disclosure, these current findings and our clinical experience also underline that the relational context and how questions are posed have an impact on the effect of probing.

As theme 2 of our overarching synthesis shows, both survivors and health professionals felt great apprehension in making CSA a shared focus of attention, because of the unpredictable consequences of doing so. Both parties, for different reasons, were torn between the felt need to address CSA and the strong feeling of not wanting to go there. This might trap the survivor and the health professional in mutual avoidance of CSA. While early, mandatory screening for CSA might support health professionals in addressing CSA, it might also constitute a potential barrier for disclosure, because the relational prerequisites for disclosure are not established. The health professionals may unknowingly protect themselves by screening in a way that follows procedures but does not invite disclosure. Providing flexibility in procedures and authorizing health professionals to apply clinical judgment thus seem paramount to facilitate increased detection rates of CSA within CAP. Moreover, clinical experience, as well as our findings, show how previous negative experiences following disclosure might interfere with efforts to detect CSA. An example of this might be how the actions of other agencies, like the legal system, impact the survivor. . Both survivors and health professionals often feel that they lose control of the situation in a very vulnerable phase for the survivor (see, also Watkins-Kagebein et al., 2019). Going forward, it is therefore important to explore how to improve collaboration between involved agencies to ensure that the best interest of the survivor is safeguarded.

Our findings also show how survivors initially engage in a process of sorting out what they have experienced and their part in it, before they are ready to purposefully disclose. In light of this it would be worthwhile to investigate further how health professionals might facilitate disclosure through supporting survivors in their exploration of their own experiences. Research also indicates that many adolescents use indirect disclosure strategies, including self-harm, or they purposefully keep the abuse a secret (Ungar et al., 2009), while clinical experience shows that a range of other difficulties commonly seen in CAP, like substance misuse, behavioural problems, avoidant behaviour, and disturbed eating may represent indirect disclosure strategies. From a clinical perspective, suspicion of CSA often emerges as a hunch based on the integration of several sources of information, with clinical judgment often determining whether we pursue the suspicion regardless of initial denial and disengagement in therapy. In these situations, health professionals typically struggle to find ways to get beyond the “dunno” and shrugs of the adolescent and to establish a relationship that can carry the relational risk of sharing CSA. Training health professionals to identify and interpret more diffuse signs that may indicate possible CSA and provide opportunities to practice how to explore and clarify suspicion of CSA in ways that are experienced as safe and benevolent by the child are therefore promising avenues to facilitate earlier detection of CSA within the context of CAP.

Ultimately, then, detection of CSA within the context of CAP is linked to the health professional’s ability to create facilitative conditions to help survivors disclose. Yet, as our findings show, disclosure is a highly complex and relational process, with high degrees of uncertainty. These uncertainties and pitfalls often lead health professionals to question their decision to pursue their suspicion. Research also shows how health professionals feel isolated in their struggles and are in need of opportunities to share their vulnerabilities and hardships from the work (Helpingstine et al., 2021; Sommer & Cox, 2005). An important clinical implication is therefore that we must offer health professionals emotional support and arenas for sharing the tolls of their work in order to equip them and safeguard them for the process of CSA disclosure. Strengthening health professionals’ sense of support and community will reduce their sense of isolation. Management should provide time and places for collegial support, including occasions for discussing clinical decisions, sharing vulnerability, and discussing how the work affects them (Albæk et al., 2019; Helpingstine et al., 2021; Sommer & Cox, 2005). As evident from the sub-synthesis, health professionals also experienced unexpected rewards from their work, and they found sharing these aspects of the work with colleagues beneficial as well. Moreover, training and structures that can reduce their feelings of inadequacy and fear of making things worse for the child (Albæk et al., 2020, 2018) are likely important measures to support health professionals in addressing CSA in ways that are helpful for the survivors.

## Scope and limitations

By integrating all available qualitative research on how children and adolescents experience the process of CSA disclosure with all available qualitative research on how health professionals experience addressing CSA, the presented synthesis contributes to the field by providing a more detailed picture of a highly complex clinical situation that is often explored from one perspective at a time. Moreover, the teams’ clinical experience is used actively to suggest clinical implications of the findings. By focusing exclusively on survivor participants interviewed before the age of 18, we were able to detail the knowledge on the disclosure process, including the initial stages of disclosure, and the significance of expanding the focus to include the consequences of disclosure for the survivor. However, all included studies on the survivor perspective were carried out in a Western context, having important implications for transferability of the findings. Future research should therefore explore the disclosure processes outside of a Western context, and include a focus beyond disclosure as the end-point of the process. The synthesis on the health professionals’ perspective represents the first of its kind, thus contributing with important knowledge on how health professionals experience addressing CSA. However, this paucity of research exploring health professionals’ perspective also means that although locating studies relevant to shed light on how health professionals experience addressing CSA, none of the included studies explicitly focused on the disclosure process as such, pointing to an important area for future research. Finally, the research team has a background from psychology/psychiatry/mental health care within a strong well-fare system. Including other researchers, e.g., from the legal system or child welfare, or from other contexts, might have contributed perspectives that might have expanded our understanding and implications of the presented findings.

## Conclusion

Making it possible for more survivors to reveal CSA earlier might be one of the most powerful secondary prevention strategies available to us. Any effort to lower the threshold for CSA disclosure within CAP that does not integrate an understanding of the immensely difficult task facing both survivors and health professionals will, however, risk failure. In our synthesis of qualitative studies on how survivors- and health professionals experience addressing CSA three themes emerged that show how both survivors and health professionals feel isolated in their struggle to restrain the destructive impact of CSA and the unpredictability and uncertainty embedded in the disclosure process and its consequences. In light of our findings, we suggest clinical implications to increase detection rates of CSA in CAP, including the importance of trusting and allowing health professionals to apply their clinical judgment, provide emotional support for professionals and opportunities for professional competence development, and ensure diverse and reflexive dialogues about CSA.

## Appendix 1

Overview and description of articles included in the meta-synthesis; 11 articles reporting on the survivors’ perspective and 14 articles reporting on the health professionals’ perspective.

**Table:**

Overview of the 11 included articles reporting on survivors’ perspective of disclosure of CSA, participants interviewed as children or adolescents					
Study	Aims	Samplea	Context	Method/design	Summary of findingsb
1. Allnock & Atkinson, 2019	Exploring school-specific barriers to victim disclosure and peer reporting of sexual harm	59 young British people aged 13–21 (32 male), average age 15	7 UK Schools	17 focus group interviews (15 single gendered), with 1–6 participants in each focus group. 12 interviews recorded and transcribed	Both social barriers, such as a hierarchy of harm, normalization of sexual harm, and a culture of not snitching, and organizational barriers, such as lack of positive relationship between students and staff, staff insensitivity in handling disclosures, and fear of escalating consequences were found.
2. Crisma et al., 2004	Understanding barriers to disclosure and needs among sexually abused adolescents	36 young Italian people (1 male), 31 < 18 years, 4 18–22 years, 1 > 22 years	Toll-free telephone line in Italy, established for the study	Anonymous, in-depth telephone interviews	Adolescents most often told friends first. 26 had never told their family, 20 of which reported a good relationship to their family. Fear of not being believed, fear of consequences for themselves and their families, and shame were barriers to disclosure.
3. Foster & Hagedorn, 2014	Exploring children’s personal perspective on CSA, disclosure and recovery	21 American children aged 6–17 (18 girls), average age 11. Ethnic diversity an aim, 24% Caucasian, 33% Hispanic, 33% African American	Large, urban child advocacy centre in the US counselling child victims of sexual abuse and their nonoffending caregiver	Analysing trauma narratives written by children as part of counselling (TF-CBT)	Survivors disclosing as children wait years before telling. All children knew their perpetrator. Betrayal of trust and sadness. Carrying the secret of the abuse was a burden. Confusion, shame, fear of not being believed, feeling responsibility for the abuse, and fear of consequences for themselves and the perpetrator were barriers for disclosure. The children experienced several negative consequences of disclosure.
4. Foster, 2017	Exploring how boys experienced sexual abuse and disclosure	19 American boys aged 3–17, average age 8.5. Ethnic diversity an aim, 42% Caucasian, 37% Hispanic, 11% African American	Large child advocacy centre in the US specialized in the treatment of abuse	Analysing trauma narratives written by boys as part of counselling (TF-CBT)	Same themes as Foster & Hagedorn, 2014. All boys knew their perpetrator but described less grooming behaviour. Initial shock and anger. Embarrassment, fear of perpetrator retaliation, worry of others’ reactions, and fear of consequences for the perpetrator barriers to disclosure. Disclosure ended abuse—yet was associated with range of strain and challenges.
5. Jensen et al., 2005	Exploring the child perspective on facilitators and barriers to CSA disclosure	22 Norwegian children aged 3–16 from 20 families. 15 girls, average age 7.5	Norwegian university clinic set up to assist families where there were serious concerns for CSA	Videos of therapy sessions and videotaped follow-up interviews with the families 1 year after the last session	Difficult for the children to initiate a dialogue about CSA. Children sensitive to the care-giver’s ability to handle the information and feared the consequences of disclosure for their families and the perpetrator. Opportunities for telling, including alone time with emotionally available caregiver, shared attention on CSA-related topics, and caregivers’ initial reactions indicating room for telling, facilitated disclosure.
6. Katz & Hamama, 2017	Exploring children’s descriptions and perceptions following alleged sibling incest	20 Israeli children aged 6–12. 17 girls, average age 9	Israeli forensic investigators exploring alleged sibling incest involving penetration	Thematic analysis of the first interview conducted as part of a forensic investigation^	Children waited years to tell. The relationship to the offender and the experience of being unable to say no to the abuse induced guilt and shame. Parents’ unavailability and rejection serious barriers to disclosure. Parents’ disbelief when finally disclosing added to the burden of the abuse.
7. McElvaney et al., 2012	Exploring the process of how children tell their experiences of CSA	22 Irish children aged 8–18. 16 girls, 21 Irish born	Irish child sexual abuse assessment and therapy service at an urban children’s hospital	Semi-structured interviews analysed using a grounded theory approach	The children actively withheld information about abuse, because of difficulties telling, not wanting others to know. In keeping the secret, the children experienced a pull between telling and not telling, with pressure building over time. When telling, this often happened in a context of shared intimacy and confidences.
8. McElvaney et al., 2014	Understand factors influencing informal disclosure of CSA	Same participants as McElvaney et al., 2012, p. 22 Irish children aged 8–18. 16 girls, 21 Irish born	Irish child sexual abuse assessment and therapy service at an urban children’s hospital	Semi-structured interviews analysed using a grounded theory approach	Disclosure was delayed, often by years. Many told peers before they told adults. Being believed, being asked, shame of what happened, blaming oneself for the abuse, concern for self and others, and peer influence influenced the disclosure process. Disclosure might have grave consequences, e.g., by splitting up families.
9. Petronio et al., 1996	Explore decision making and strategies used by children to disclose CSA	38 American children aged 7–18 (6 boys) who had willingly disclosed the abuse to family, peers, or friends	An US non-profit agency	Face-to-face interviews, analysed using Communication Management of Privacy Theory as a framework	Children used boundary access rules, such as tacit permission, choosing circumstance, and incremental disclosure when sharing their experiences. They also protected their privacy using boundary protection rules, such as anticipating consequences of disclosure and evaluating potential disclosure recipients.
10. Shalhoub-Kevorkian, 2005	Explore the transferability and effect of Israelian social politics to Palestinian Israeli children	28 Palestinian Israeli girls aged 14–16	An Israeli nongovernmental organization: Women Against Violence	Interviews and focus groups, analysed with a grounded theory approach	Barriers for disclosure included fear of losing love and support from their families, shame, self-blame, fear of violent retaliation, fear of destroying their own and their family’s reputation, and eliminating opportunities for their future.
11. Staller & Nelson-Gardell, 2005	Enhance understanding of the process of CSA disclosure from the perspective of young people	34 American girls aged 10–18. Average age 13.7, 70% White	Various US agencies offering therapy and counselling to child and adolescent victims of sexual abuse	Secondary analysis of focus group interviews with 4 treatment groups for girls who had experienced CSA. 5–10 participants in each focus group.	Disclosure process three phases: 1) ‘Self’, where the survivors have to understand what they have been exposed to; 2) ‘Confidant selection-reaction’, where survivors chose disclosure recipient and 3) ‘Consequences’, where initial reactions keep informing survivors’ on-going strategies of telling.
**Overview of the 14 included articles reporting on health professionals’ perspective of addressing CSA**					
**Study**	**Aims**	**Sample**a	**Context**	**Method/design**	**Summary of findings**b
1. Benatar, 2000	Explore effect of own history of CSA among experienced therapists treating survivors of CSA	6 American therapists with history of CSA and 6 American therapists without such experiences. Same age span (24–57) and level of experience (7–35 years) in the two groups. 2 men	Trauma therapists working with adult survivors of CSA in the US	In-depth interviews	No differences between the two groups. Working with survivors of CSA affected therapists—positively and negatively. Five areas of vicarious trauma were identified (world view, sense of safety, relationship to work, relationship to self, and relationship with others). Five related themes of positive self-transformation were also identified (self-esteem/ empowerment, mind expansion/ wisdom, work with other clients, validation/healing, and activism).
2. Capri et al., 2013	Explore emotional impact of health professionals working with survivors of CSA	4 White South-African CSA Workers aged 27–38, all female. 4–10 years experience of working with CSA	South-African non-profit organization treating children who have experienced CSA	7 individual, semi-structured interviews analysed with thematic analysis	The strain of working with CSA, and the significance of inner resources and support when working with CSA for the work to be sustainable. The therapists held a deep respect of the cost of the work and were very attentive to signs of burn-out and overidentification, while maintaining the significance of finding the balance allowing for empathic involvement.
3. Engh Kraft et al., 2017	Explore the ability of school nurses to detect and support sexually abused children	23 female, Swedish school nurses, aged 46–67, with 3–38 years of experience	Swedish school nurses	Secondary analysis of 8 focus group interviews with 4 groups of school nurses. 4–6 participants in each group. Thematic analysis used	The complexity of the disclosure process, the strong emotions potentially elicited, and a sense of vulnerability due to working alone keeps the school nurses from addressing CSA. Yet they feel an obligation to do so and use different strategies to help the child open up. Tension between blaming themselves for not seeing CSA and not wanting to see CSA.
4. Gallop et al., 1995	Explore opinions of nurses with and without a history of CSA of routine inquiry of CSA	323 American nurses working in diverse practice settings, age range 31–68. 42 of the participants had a history of CSA.	Nurses in diverse practice settings in the US. All from the same metropolitan area.	Written answers to open questions in anonymous questionnaire and individual interviews with at least 6 nurses with a history of CSA (no accurate information provided)	No clear difference between the two groups. The majority positive towards routine inquiry of CSA in paediatric and psychiatric settings but felt insecure regarding their own competence. Those not favouring routine inquiry stressed the potential of increasing client distress, robbing survivors of control, as well as their own feeling of inadequacy in handling the emotional intensity if CSA was detected.
5. Jensen et al., 2010	Exploring what constitutes a good alliance in therapies with children that may have been sexually abused	5 Norwegian psychologists with more than 10 years experience of working with children	Norwegian therapists working with children referred to psychotherapy because of suspicion of CSA	Therapists’ reflective notes following each session for 15 cases, focusing on what difficulties therapists had encountered, and how they felt about the relationship to the child and the caregiver	The significance of the relationship between therapist and caregiver to ensure a good relationship with the child. Therapist experienced uncertainty regarding when and how to introduce difficult topics. Therapists actively used caregivers’ reactions and signs to adjust their work and to time their interventions.
6. Possick et al., 2015	Understanding the dialectic between the destructive forces of trauma and forces that foster growth in trauma therapists	14 experienced Israeli social workers (1 male) aged 36–49 with 6–28 years of relevant work experience	Israeli social workers treating children exposed to CSA in government or non-profit therapy centres	In-depth semi structured interviews analysed with IPA	Dialectic between two emotional poles, where the first pole shed light on how relating to CSA destroys the experience of trust and order in the therapists’ familiar and secure world. The other pole shed light on how clinical and moral activity on behalf of the child restores therapists’ faith in recovery and growth.
7. Sekhar et al., 2018	Explore experiences of reporting suspected CSA and impression of a new screening tool	17 American school nurses (1–29 years of experience), 6 American school counsellors (5–20 years of experience), 14 paediatricians (10 months-25 years of experience)	Key stake holders for CSA detection in a US context. All from the same state	2 focus group interviews with school nurses, 2 focus group interviews with paediatricians, 2 focus group interviews with schoolteachers, counsellors and administration (only counsellors included in the analyses)	Children get better at hiding abuse as they get older. The significance of early screening for CSA and education to help children distinguish safe from unsafe touch. Several dilemmas of screening in different settings, particularly challenges regarding confidentiality and how to detect CSA when parents are the perpetrator. Better to provide recurrent opportunities to tell rather than asking directly and concerns how recurrent questions of CSA could impact children.
8. Sivis-Cetinkaya, 2015	Explore school counsellors’ experiences of reporting CSA	25 Turkish school counsellors who had reported CSA the past 5 years. 7 male	Turkish school counsellors	Content analysis of written responses to open questions in an anonymous online survey	Mixed experiences and emotions in reporting CSA: An emotional cost, including fear of retaliation from victim’s family, sadness, and felt helplessness, but also positive emotions, including relief, courage, and strength. Colleagues, lawyers, and principals were important sources of support, but also potential barriers.
9. Slane et al., 2018	Explore different professions’ view of the usefulness and cost of viewing images of CSA as part of their work with survivors	35 Canadian child mental health professionals. No further information provided	Canadian child and adolescence mental health care system. All participants from the same state	5 focus group interviews with child mental health professionals	None of the CMH professionals felt they needed to see images of the abuse to do their job. The necessity to find the balance between self-care and protecting oneself from unwarranted strain, while taking in and relating to what CSA really is. How relating to CSA changes relations to others
10. Steed & Downing, 1998	Exploring how relating to trauma content influences trauma therapists working with sexual abuse/assault survivors	12 female, Australian trauma therapists aged 26–59 with 1–18 years of experience working with sexual abuse/assault survivors	Australian agencies working with survivors of sexual abuse and sexual assault. Age of clients unknown. All participants from the same area	Semi-structured, individual interviews analysed with thematic content analysis	The work affected the therapists negatively outside work, including increased anger an insecurity, and relational difficulties. All reported having lost faith in humankind and an increased sense of vulnerability. The work also impacted their sense of identity negatively. At the same time they have witnessed the strength of their clients, and how resourceful people are. Therapists underlined the significance of self-care.
11. Sui & Padmanabhanunni, 2016	Explore therapists’ experience of working with trauma survivors and the psychological consequences of working with trauma	3 female South African therapists with >3 years experience of working with sexual abuse/sexual trauma	South African non-governmental organizations. Age of clients unknown	Individual semi-structured interviews analysed with thematic analysis	The work took its toll, including therapists experiencing a sense of isolation from others, intrusive memories, somatic symptoms, and unwanted changes in their way of being. However, the work was also associated with positive transformation, including personal growth and healing, and a sense of wisdom.
12. Søftestad & Toverud, 2013	Exploring how social workers experience working with families where they suspect CSA	11 Norwegian social workers (1 male)	Norwegian child protective services. All participants worked in the same area	Individual interviews analysed with a grounded theory approach	The social workers felt inadequately trained to address CSA and felt a need for more direct contact with the children to do better clinical judgements. High workloads and the range of different work tasks hindered expertise development. The importance of support from colleagues and leadership was stressed.
13. Wheeler & McElvaney, 2018	Exploring the positive impact of working with children who have been sexually abused	9 female Irish trauma therapists aged 36–65 (average 49), affiliated with the organization for 1–20 years (average 6).	An Irish organization that specializes in providing therapy to children who have been sexually abused	Unstructured interviews analysed using an inductive thematic analysis	Unfamiliar focus for therapists but experienced as fruitful. They experienced a professional satisfaction in being able to help children get a better life—a sense of saving lives. Working with the children was also a learning experience, strengthening their attitudes and life philosophy, and helping them develop traits such as creativity and honesty. The connection with the children during sessions was an important source of joy contributing to making the work sustainable.
14. Överlien & Hyden, 2003	Explore and analyse how staff reflect and talk together when facing a situation where they suspect sexual abuse	No information provided beyond general characteristics of the staff: 11 resident assistants, two half-time psychologists, a part-time medical doctor, a full-time nurse	Swedish detention home, specifically staff at a ward for six young women of 16–19 years, who had been viewed by the Social Services Department as having complex psychosocial problems	Narrative analysis of five group interviews and 29 individual interviews in relation to five concrete situations where staff has encountered a dilemma in relation to suspected sexual abuse. Collected as part of ethnographic field work	The narrative analysis shed light on the conceptualization of ‘must-be-told-stories’, where disclosure is regarded necessary for recovery, and staff consequently should work actively to facilitate disclosure, as opposed to ‘cannot-be-told-stories’, where putting the story of abuse out in the open can lead to catastrophic consequences, both for the client, and sometimes also the staff. This conceptualization warranted the staff to approach the clients and potential stories of abuse with great care and precise clinical judgment.

aFor studies including different groups of participants, only the sample included in the meta-syntheses is described

bFor studies with a broader scope, only those parts of the findings relating to the disclosure process/addressing CSA have been included in the syntheses ^ Included even though used forensic interview as data because of the open format of the interview, the high degree of transparency of the study, and the numerous quotes included
